# Thalamic functional connectivity and sensorimotor processing in neurodevelopmental disorders

**DOI:** 10.3389/fnins.2023.1279909

**Published:** 2023-12-15

**Authors:** Smitha Karavallil Achuthan, Despina Stavrinos, Paula Argueta, Caroline Vanderburgh, Haley B. Holm, Rajesh K. Kana

**Affiliations:** ^1^Department of Psychology and the Center for Innovative Research in Autism, The University of Alabama, Tuscaloosa, AL, United States; ^2^Department of Psychology and the Institute of Social Science Research, The University of Alabama, Tuscaloosa, AL, United States; ^3^Children’s Hospital of Atlanta, Atlanta, GA, United States

**Keywords:** ADHD, autism, thalamus, fMRI, resting-state, connectivity

## Abstract

One of the earliest neurobiological findings in autism has been the differences in the thalamocortical pathway connectivity, suggesting the vital role thalamus plays in human experience. The present functional MRI study investigated resting-state functional connectivity of the thalamus in 49 (autistic, ADHD, and neurotypical) young adults. All participants underwent structural MRI and eyes-open resting state functional MRI scans. After preprocessing the imaging data using Conn’s connectivity toolbox, a seed-based functional connectivity analysis was conducted using bilateral thalamus as primary seeds. Autistic participants showed stronger thalamic connectivity, relative to ADHD and neurotypical participants, between the right thalamus and right precentral gyrus, right pars opercularis-BA44, right postcentral gyrus, and the right superior parietal lobule (RSPL). Autistic participants also showed significantly increased connectivity between the left thalamus and the right precentral gyrus. Furthermore, regression analyses revealed a significant relationship between autistic traits and left thalamic-precentral connectivity (R^2^ = 0.1113), as well as between autistic traits and right postcentral gyrus and RSPL connectivity (R^2^ = 0.1204) in autistic participants compared to ADHD. These findings provide significant insights into the role of thalamus in coordinating neural information processing and its alterations in neurodevelopmental disorders.

## Introduction

Thalamus, one of the functional hubs of the brain, is primarily involved in relaying sensory information, modulating motor signals, and regulating consciousness (Hwang et al., 2017; Torrico and Munakomi, 2023). It is composed of extensive nuclei that provide connections over the entire cerebral cortex. These nuclei are either first-order, receiving input from ascending sensory pathways, or higher order, receiving input from the cortex. However, the notion that the thalamus solely serves as a passive relay station underestimates the substantial capabilities of this key structure in higher-order information processing. The anterior nuclei of the thalamus have been implicated in visual and verbal memory tasks, temporally before the hippocampus (Štillová et al., 2015). As previously reported, the thalamus has a role in monitoring and maintaining mental constructs (Wolff and Vann, 2019) and in language processing, especially linguistic procedural memory (Crosson, 2021). The medial dorsal thalamus, on the other hand, has been implicated in executive functioning due to its interconnectivity with the prefrontal cortex (Ouhaz et al., 2018). Hence, it is likely that any neurological or neurodevelopmental condition associated with structural and functional differences in the thalamus may accompany some of the symptoms commonly seen in autism spectrum disorder.

In addition to social communication difficulties and repetitive behaviors, autism is characterized by hyperreactivity or hyporeactivity to sensory information (Hodges et al., 2020). Considering extensive reports of behavioral, sensory, motor, and social differences seen in autistic individuals, it is quite possible that many of the neurobiological differences in autism on deeper inquest may lead to differences in anatomical organization and functional engagement of the thalamus. Although no significant volumetric differences in thalamus have been reported in autism (Edgar et al., 2015), the right posterior thalamus has shown some differences, such as a more expanded surface area, and a more concave shape for the left mediodorsal nucleus (Schuetze et al., 2016). Mouse studies mimicking human phenotypes in a monogenic form of autism have found that thalamic reticular nucleus (TRN)-mediated thalamic gain control may underlie perceptual and attentional differences seen in autism. This has led to the “leaky thalamus” hypothesis, which states that altered thalamic regulation allows irrelevant sensory information to bypass the thalamus and become more distracting. This imbalance in thalamic regulation could underlie differences in information processing in autism (Schmitt and Halassa, 2017). Furthermore, irregularities in thalamic neurochemistry can prevent thalamic ability to inhibit sensory information, leading to sensory over-responsivity (SOR). For instance, a recent study on the effects of neurotransmitters like the Gamma Aminobutyric Acid (GABA) and glutamate found that thalamic GABA in autistic participants predicted altered functional connectivity in regions implicated in SOR. Thus, thalamic neurochemistry differences in autism could interfere with selective attention to sensory stimuli (Wood et al., 2021), which, in turn, can lead to a cascade of higher order processing challenges.

Thalamic resting-state functional connectivity networks (RSN) in neurotypical brains are believed to be inhibitory in nature. As a relay station for transmitting information across cortical networks, the thalamus works to inhibit irrelevant and obstructive sensory input (Tang et al., 2011). In autism, fMRI studies have reported altered thalamic activation and connectivity patterns across cortical networks. The existing literature suggests patterns of hyperconnectivity during resting state between the thalamus and several other brain regions in autism. A recent study, using a relatively large sample of 368 autistic and 362 neurotypical participants from the Autism Brain Imaging Data Exchange (ABIDE), reported hyperconnectivity in autism between the thalamus and 19 cortical regions, including the frontoparietal cortices, left temporoparietal junction (LTPJ), and the bilateral posterior cingulate cortex (PCC; Iidaka et al., 2019). Another study on autistic adults found hyperconnectivity between the right thalamus and bilateral precentral and postcentral gyri, and right superior parietal lobule (RSPL; Ayub et al., 2021). Yet studies, examining autistic children and young adults (ages 8–25 years), found hyperconnectivity between the thalamus and the temporal cortex, suggesting less filtered relay of auditory information (Baran et al., 2023) and hyperconnectivity with limbic and sensorimotor regions perhaps caused by early overgrowth in these regions in autism (Nair et al., 2015). Temporal and motor thalamocortical hyperconnectivity have also been reported in autism, including the somatosensory cortex and prefrontal cortex (Woodward et al., 2017). Studies examining infants and young children found significant hyperconnectivity between the thalamus and occipital and motor regions (Nair et al., 2021), and between the thalamus and auditory cortex, which was found to be associated with sensory and sleep difficulties (Linke et al., 2023). Despite the differences in specific brain regions found in each study, there is a common occurrence of increased connectivity in thalamic RSN in autism. This may lead to increased and less filtered sensory information being relayed to the cortex, which may alter sensory processing in general.

Another neurodevelopmental disorder, attention-deficit hyperactivity disorder (ADHD) is characterized by hyperactivity, inattention, and impulsivity in childhood with a risk of persisting into adulthood (Wolraich et al., 2019). Despite the increased prevalence of ADHD, there is limited research on thalamic connectivity in ADHD. This may be due to several factors including, but not limited to, the specific symptomatology and the impact of commonly used ADHD medications on MRI signal and functional connectivity. The role of the thalamus in ADHD symptomatology is widely neglected, despite having significant effects on arousal and motivation, as demonstrated by EEG data (Bailey and Joyce, 2015). The thalamus is implicated in attentional control by linking memory and visual focus (de Bourbon-Teles et al., 2014) and mediating arousal and selective attention (Portas et al., 1998). The thalamus also plays a role in impulsivity through modulating inhibitory control (Halassa and Acsády, 2016; Wei and Wang, 2016). Furthermore, studies have shown that the most common prescription medications to treat ADHD, such as methylphenidate and amphetamine (Subcommittee on Attention-Deficit/Hyperactivity Disorder, Steering Committee on Quality Improvement and Management et al., 2011; Wolraich et al., 2019) can alter thalamocortical connectivity in children and adults with ADHD (Avram et al., 2022; Kaiser et al., 2022).

The majority of resting-state connectivity studies on the thalamus in ADHD focus on cortico-striato-thalamo-cortical (CSTC) loops (Posner et al., 2014). A recent study reported a positive correlation between thalamocortical connectivity with the frontoparietal control network and severity of hyperactive or impulsive symptoms as measured by the ADHD Rating Scale-IV (Hong, 2023). Another study found hyperconnectivity between the thalamus and the left putamen, which is involved in higher-level cognitive processes, and abnormal connectivity between the putamen and CSTC loops in ADHD participants who remained medication-naïve (Cao et al., 2009). Dysconnectivity between the thalamus and striatum was reported to be caused by disrupted white matter fiber connections in children with ADHD (Xia et al., 2012). Yet another study on children with ADHD found hyperconnectivity between the occipital and parietal thalamic region of interest and the left putamen and right caudate head, which is implicated in spatial span working memory (Mills et al., 2012).

Thus, considering the potential role of thalamus in neurodevelopmental disorders in general, the primary goal of the current study is to examine functional connectivity in autism and ADHD by focusing on the thalamus as the seed. Based on previous findings of hyperactivation and connectivity patterns of the thalamus with several brain regions, we predict greater thalamic connectivity to motor, somatosensory, and visuospatial areas in autistic, compared to NT and ADHD, participants during resting state fMRI. The findings of this study will provide significant insights into alterations in thalamic connectivity in neurodevelopmental disorders. We also predict that a greater presence of autistic symptoms, measured by the autism spectrum quotient (AQ), will have a positive relationship with thalamic connectivity. This is based on previous findings that autistic traits such as sensory modulation difficulties most likely involves functional alterations in thalamocortical connectivity, as the thalamus is known to connect primary sensory input with higher-order cortical areas.

## Materials and methods

### Study participants

The current study includes 49 young adults (n_autistic_ = 15; n_adhd_ = 19; n_neurotypical_ = 15). The following criteria were used for inclusion of participants: participants’ age between 16 and 30 years, full-scale intelligence quotient (FSIQ) of 70 or higher, a self-reported clinical diagnosis of ASD and a cut-off score of 26 or higher on the AQ for autistic participants (Baron-Cohen et al., 2001), a self-reported clinical diagnosis of ADHD and a score of 4 or higher on the Adult ADHD Self Report Scale (ASRS) short-form (Kessler et al., 2005). Participants that self-reported a comorbid diagnosis of ASD + ADHD were excluded from the current study; thus, none of the ASD participants in this study had co-occurring ADHD. Participants taking stimulant medications were requested to withhold taking their medication 24 h prior to their scans (100% compliance reported). Participants were excluded from the study if they had a history of a severe mental disorder (e.g., bipolar, schizophrenia), intellectual disability, obsessive-compulsive disorder, Tourette’s syndrome, epilepsy, traumatic brain injury, or concussion with loss of consciousness. Participants currently using antipsychotics, anticonvulsants, benzodiazepines, or chemotherapy agents were excluded. Current psychotropic prescription medication use was recorded for each participant. Medications included stimulants (n = 10), norepinephrine-dopamine reuptake inhibitors (NDRI; *n* = 4), selective serotonin reuptake inhibitors (SSRI; *n* = 7), selective norepinephrine reuptake inhibitors (SNRI; *n* = 3), anxiolytics (*n* = 1), alpha 2a adrenergic receptor agonist (*n* = 1), and histamine H1 agonist (*n* = 1). No psychotropic medication use was reported in the TD group. Several participants were currently taking more than one psychotropic medication (*n* = 6). Detailed demographic information is provided in Table 1.

**Table 1:** Participant demographic information.

	Autistic mean ± SD (range)	NT mean ± SD (range)	ADHD mean ± SD (range)	Autistic vs. ADHD (*p*)	Autistic vs. NT (*p*)	ADHD vs. NT (*p*)
Age	19.2 ± 2.2 (16–22)	20.8 ± 3.6 (16–29)	19.9 ± 3.2 (16–26)	0.23	0.24	0.85
Gender	9 male; 6 female	9 male; 6 female	11 male; 8 female			
FSIQ	108.7 ± 20.05 (65–142)	104.7 ± 10 (79–120)	108.1 ± 10 (85–131)	0.041	0.052	0.848
SRS-2 total	144.2 ± 24.2 (107–174)	104.9 ± 7.2 (91–117)	121.9 ± 15.6 (98–152)	0.01	0.0001	0.002
AQ RAW SCORE	33.3 ± 7.7 (26–47)	13.9 ± 5.5 (5–25)	20.1 ± 4.0 (12–28)	0.0000003	0.0006	0.00000001
Max head motion (mm)	1.4 ± 1.8 (0.251–7.49)	1.9 ± 3.9 (0.337–15.45)	2.2 ± 2.5 (0.382–9.54)	0.272	0.260	0.650
Mean head motion (mm)	0.13 ± 0.04 (0.07–0.26)	0.13 ± 0.03 (0.092–0.203)	0.15 ± 0.04 0.095–0.22	0.528	0.299	0.020
Framewise displacement (mm)	0.19 ± 0.07 (0.101–0.386)	0.19 ± 0.06 (0.123–0.323)	0.22 ± 0.06 (0.125–0.365)	0.648	0.733	0.333

All participants underwent structural MRI and resting state fMRI scans on a Siemens 3 Tesla Prisma magnet. We used a 20-channel phased array head coil for imaging. The structural data were acquired using T1-weighted MPRAGE (magnetization-prepared rapid gradient echo) sequence with a TR (repetition time) =2,400 ms; TE (echo time) =2.22 ms; FOV (field of view) = 256 mm; matrix size = 208 × 300 × 320, ascending interleaved acquisition yielding 208 transverse slices, voxel size = 0.8 × 0.8 × 0.8 mm^3^; slice thickness = 0.08 mm. inversion time = 1; flip angle = 8°; bandwidth = 220 Hz/Px, PAT factor = 2 (GRAPPA mode). The resting fMRI data were acquired using a multiband sequence involving two acquisitions adding up to 10.92 min. One acquisition was in the anterior to posterior direction (A > P) and other in the posterior to anterior direction (P > A) by using a multiband echoplanar imaging sequence each 5.46 min long. The imaging protocol involved TR = 800 ms; TE = 37 ms; flip angle =52°, bandwidth = 2,290 Hz/Px, FOV = 208 ms; multiband acceleration factor = 8; interleaved multi acquisition mode; slice thickness = 2.0 mm, voxel size = 2.0 × 2.0 × 2.0 mm; yielding 72 transversal slices. During resting fMRI, participants were instructed to keep their eyes open and look at a white fixation cross on a black background which was presented on an MR-safe BOLD ^++^ screen. The image processing was done in Conn Connectivity toolbox (Whitfield-Gabrieli and Nieto-Castanon, 2012) and Statistical Parametric Mapping (SPM12) software (Wellcome Trust Centre for Neuroimaging; http://www.fil.ion.ucl.ac.uk/spm). The study used Conn toolbox’s default preprocessing pipeline for volume-based analyses. All participants’ functional images were realigned and unwarped and head motion outliers were identified. The head motion parameters created after realignment procedure were taken as a first level covariate. The functional and structural images were normalized onto a standard MNI (Montreal Neurological Institute) space, and the structural images were then segmented into gray matter, white matter, and cerebrospinal fluid (CSF; Ashburner and Friston, 2005). The spatially normalized images were subjected to smoothing using a spatial convolution Gaussian kernel of 5 mm full width at half maximum (FWHM). A band pass filter of 0.008–0.09 Hz was applied to the smoothed data. Denoising was achieved using a procedure called *aCompcor* (anatomical component-based noise correction procedure). The *aCompcor* corrects for physiological noise by regressing the white matter and CSF (Behzadi et al., 2007) parameters derived from outlier scans or scrubbing (Friston et al., 1996), six head motion parameters (x, y, and z directions of translation; and pitch, roll, and yaw angles of rotation) and their first order derivatives of realignment. The maximum head motion, mean head motion, and framewise displacement are shown in Table 1.

### Seed-to-voxel connectivity analysis

To examine the resting state fMRI functional connectivity across the autistic, ADHD, and NT participants, seed-to-voxel correlation analysis was performed for two seed regions, the right and left thalamus. The left and right thalamic regions were chosen to help further explain the relationship between thalamocortical connectivity and sensorimotor function, restricted/repetitive behaviors, and somatosensory input. The average time course of resting fMRI BOLD signal from right and left thalamus was extracted and correlated with the time course of voxels from the rest of the brain to obtain the Pearson correlation coefficients. Those with a statistically significant higher and lower correlation were displayed as a functional connectivity map. The seed-to-voxel connectivity between autism and ADHD, autism and NT, and ADHD and NT were analyzed by using two-sampled t-tests. The results were statistically constrained using a voxel level threshold of *p* = 0.001 (uncorrected) and cluster level threshold of *p* = 0.005 (familywise error corrected). The functional connectivity maps for the seed ROIs (right and left thalamus) for autism > ADHD, autism > NT, and ADHD > NT were created using conn functional connectivity toolbox. A linear regression graph was plotted for each of the significant clusters with AQ raw cut off scores of autism and ADHD.

## Results

**Figure 1:**
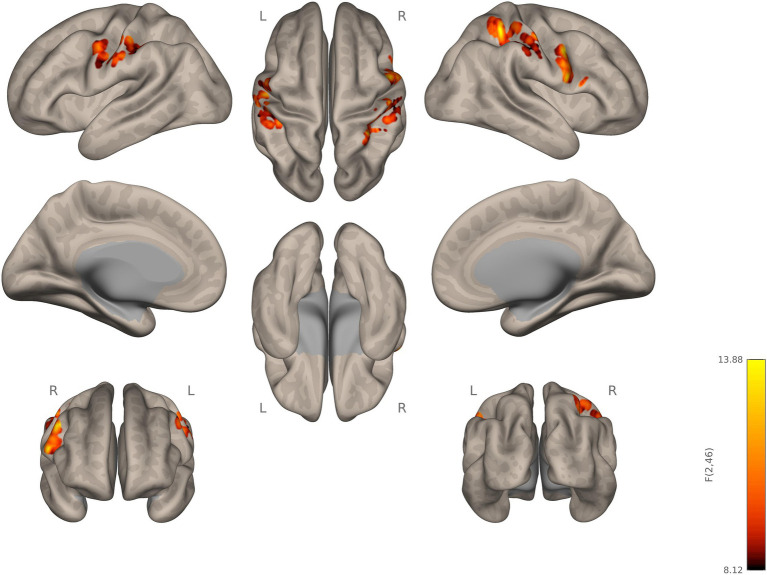
Seed-to-voxel connectivity of right and left thalamus. Autistic participants showing hyperconnectivity compared to ADHD participants.

The group comparison of functional connectivity showed statistically significant increase in connectivity in autistic, compared to ADHD, participants between the right thalamus and right precentral gyrus, right pars opercularis-BA44, right postcentral gyrus, and RSPL. The left thalamus in autistic participants showed significantly increased connectivity in the right precentral gyrus relative to ADHD. Thus, autistic participants showed stronger connectivity, relative to ADHD, of both left and right thalamus as the seed ROI with several other regions (see Figure 1; Table 2).

**Table 2:** Group differences in Thalamic connectivity across autism, NT, and ADHD participants.

Seed	Contrast	MNI Coordinate	Peak regions	Cluster size	*t*-value
*Right thalamus*	Autism > ADHD	+58 + 10 + 18	Right precentral gyrus and right pars opercularis-BA44	92	5.37
		+44 −32 + 56	Right post central gyrus and right superior parietal lobule	86	5.79
*Left thalamus*	Autism > ADHD	+56 + 00 + 44	Right precentral gyrus	81	5.68
*Right thalamus*	Autism > NT	+58 + 06 + 28	Right post and precentral gyrus, right superior parietal lobule, right anterior supramarginal gyrus, right inferior frontal gyrus pars opercularis	1,374	7.79
		−52 −28 + 42	Left pre and post central gyrus, left Supramarginal gyrus, left parietal operculum cortex, left central opercular cortex	816	6.96
		+60 −02 −04	Right central opercular cortex, right superior temporal gyrus	76	5.08
*Left thalamus*	Autism > NT	−40 −76 + 4	Left inferior lateral occipital cortex	75	6.32

**Figure 2:**
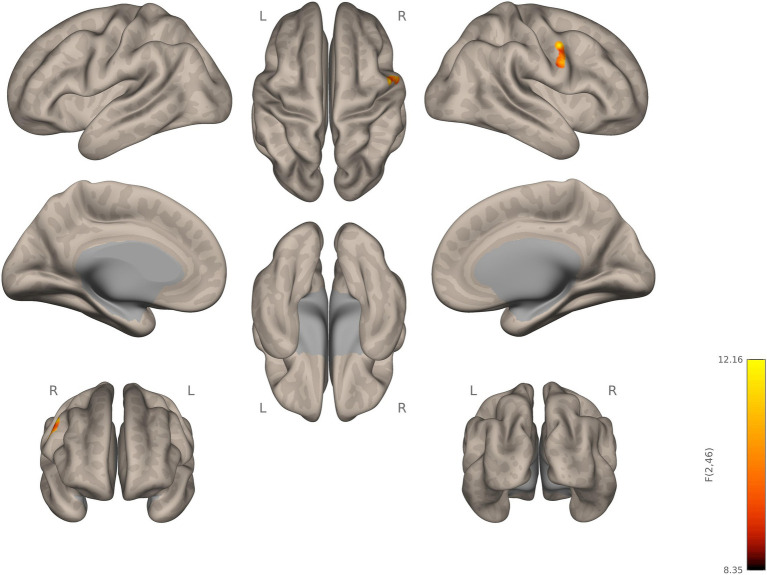
Seed-to-voxel connectivity of right and left thalamus. Autistic participants showing hyperconnectivity compared to NT participants.

The two-sampled t-test for the contrast; autism > NT, with right thalamus as the seed, showed statistically significant hyperconnectivity for autistic, relative to NT, in the right post and precentral gyrus, RSPL, right anterior supramarginal gyrus, right inferior frontal gyrus pars opercularis, left pre and post central gyrus, left supramarginal gyrus, left parietal opercular cortex, bilateral central opercular cortex, and the right superior temporal gyrus (RSTG). For the left thalamus as a seed, autistic participants, relative to NT, showed statistically significant increase in connectivity in the left inferior lateral occipital cortex (see Figure 2; Supplementary Figure S1).

**Figure 3:**
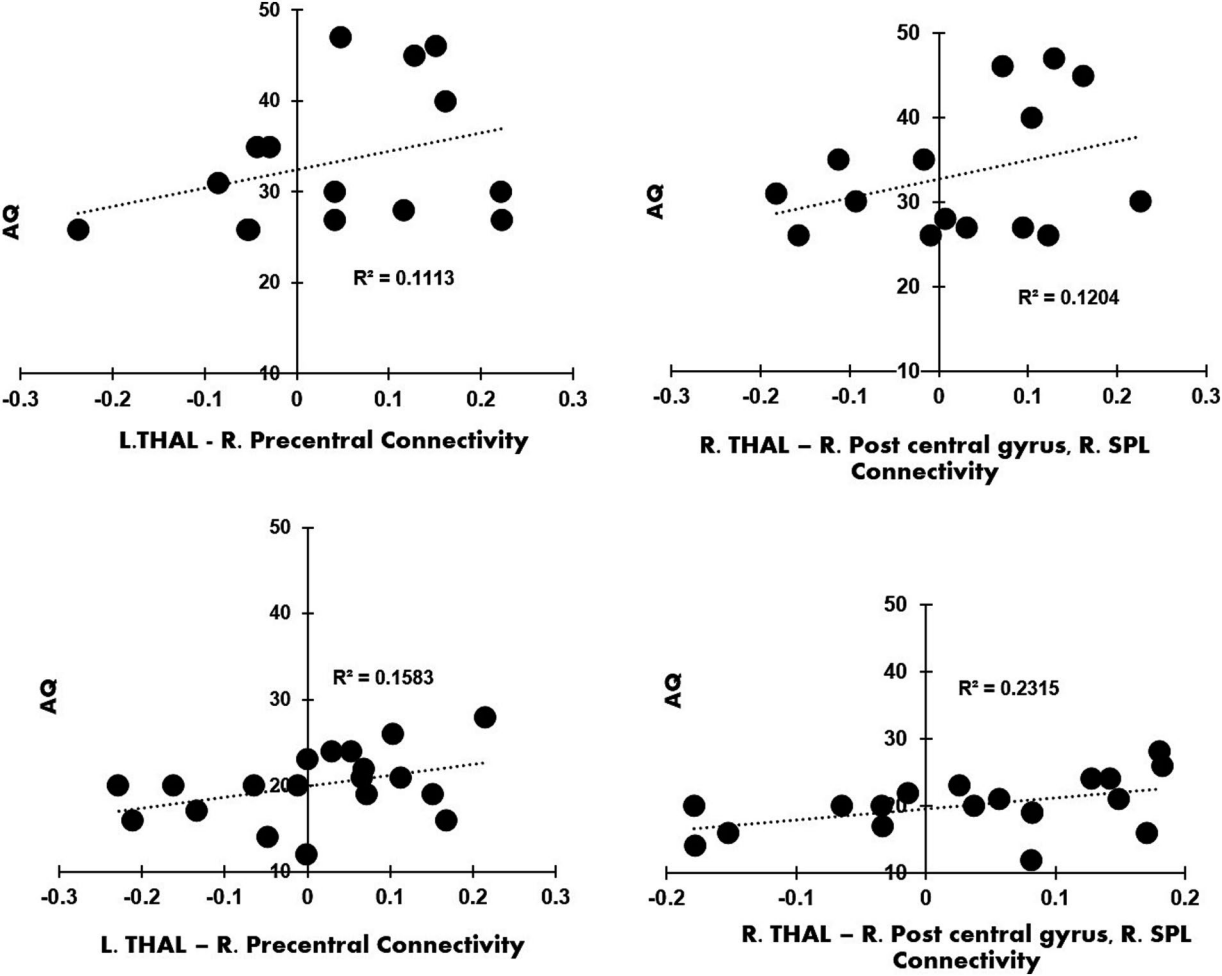
**(A)** Contrast Autism > ADHD, left thalamus as seed: linear regression plot of the connectivity values from the right precentral gyrus with the AQ raw cut off scores from autistic participants; **(B)** the contrast Autism > ADHD, right thalamus as seed; the linear regression plot of the connectivity values from right post central gyrus and right superior parietal lobule with the AQ raw cut off scores from autistic participants; **(C)** the contrast Autism > ADHD, left thalamus as seed: linear regression plot of connectivity values from right precentral gyrus with the AQ raw cut off scores from ADHD participants; **(D)** shows the contrast Autism > ADHD, right thalamus as seed: linear regression plot of connectivity values from right post central gyrus and right superior parietal lobule with the AQ raw cut off scores from ADHD participants.

The seed-to-voxel analysis with right and left thalamus did not result in any significant clusters for the contrast, ADHD > NT. Connectivity values from the clusters were obtained from the contrast autism > ADHD and autism > NT for left and right thalamus seeds. The regression analyses revealed a relationship that trends toward significance between autistic traits and left thalamic-precentral gyrus connectivity (R^2^ = 0.1113). The regression analyses also revealed a relationship that trends toward significance between autistic traits and right thalamic-right postcentral gyrus and RSPL connectivity (R^2^ = 0.1204) in autistic participants compared to ADHD. We plotted a linear regression graph for each of the clusters with AQ raw cut off scores for autism and ADHD (see Figure 3). Between subject 2 × 2 ANOVA was performed to analyze the simple effects of the three groups (autistic, ADHD, NT), with left and right thalamus as the seed regions and by controlling the mean head motion with a voxel threshold, *p* = 0.001 (uncorrected); cluster threshold, *p* = 0.005 (familywise error corrected). Peak regions obtained were presented in Supplementary Table S1 and Supplementary Figure S2.

## Discussion

The main finding of this study is a significant thalamic hyperconnectivity in autistic young adults, but not in young adults with ADHD. The finding of thalamic hyperconnectivity in autistic young adults is consistent with the literature. There have been extensive reports on hyperconnectivity between the thalamus and various other cortical regions in autistic adults (Nair et al., 2015; Ayub et al., 2021; Baran et al., 2023). A relatively recent study using ABIDE data found that thalamocortical hyperconnectivity was correlated with pathophysiological changes in autistic young adults (Iidaka et al., 2019). Another study suggested that autism is characterized by increased connectivity between the thalamus and temporal, somatomotor, and parietal regions (Hwang et al., 2021).

Other studies have reported hypoconnectivity in thalamocortical regions in ADHD. A previous study examining rs-fMRI scans of young children with ADHD found decreased connectivity between the thalamus and the right precentral, bilateral lingual, bilateral occipital, and the fusiform areas (Mills et al., 2012). Furthermore, another study found reduced activation in the thalamus, caudate, and the bilateral inferior prefrontal cortex, and the severity of ADHD symptoms were negatively correlated with the extent of activation (Cubillo et al., 2010). There are only a few studies that compare the functional connectivity profiles in ADHD and autism (Jung et al., 2019; Albajara Sáenz et al., 2020). Among them, fewer investigate thalamic resting state connectivity. One such study focused on motor inhibition found reduced inferior fronto-insular-thalamic connectivity in young autistic and ADHD adults during failed inhibition. However, no connectivity differences were found between those in separate autistic or ADHD groups (Lukito et al., 2023). In the following paragraphs, we discuss the connectivity profiles in ADHD and autism evident from the present study.

We found hyperconnectivity of the thalamus to cortical areas such as the somatosensory cortex, motor cortex, and SPL in our autistic participants. The post central gyrus has been found to have a role in perceiving somatic sensations from the body including touch, pressure, temperature, and pain (DiGuiseppi and Tadi, 2023; Raju and Tadi, 2023). The precentral gyrus, where the primary motor cortex is located, is central to the control of voluntary motor movement, and the SMA in planning voluntary limb movement (Banker and Tadi, 2023). The SPL, which is also part of the default mode network (DMN), has been found to be involved in mental imagery, recalling personal experiences, daydreaming and introspection (Cieri and Esposito, 2018) as well as in attention, visuospatial perception, and manipulation of objects (Koenigs et al., 2009). These results highlight the previous findings of the key role of thalamocortical pathways in sensory processing and perception, especially in carrying information from primary sensory organs to various cortical areas. Thus, the overall connectivity profile evident from the current study point to strong relationships of thalamus with visuospatial, motor, and somatosensory areas within autism.

While both left and right thalamus in autism have shown increased connectivity with right precentral/motor areas, the right thalamus, especially, was strongly connected to the right pars opercularis, post central, primary motor, and superior parietal areas. Hyperconnectivity within these regions may lead to suboptimal recruitment of neural networks, which, in turn, can affect the quality of behavioral engagement (Sours et al., 2015; Hillary and Grafman, 2017; Baran et al., 2023). A previous fMRI study found disrupted thalamic connectivity with bilateral pre and postcentral gyrus and LSPL in autistic adolescents when responding to aversive auditory and tactile stimuli, suggesting difficulties with filtering and/or integrating sensory information (Green et al., 2017). Another study from our own group found that autistic individuals with a greater instance of restricted interests and behaviors showed reduced recruitment of SPL during motor learning (Travers et al., 2015). In general, large-scale cortical–subcortical connectivity differences have been implicated in social communication and repetitive behaviors seen in autism (Fu et al., 2019; Tian et al., 2022). Specifically, Fu et al. (2019) reports different patterns of resting state functional connectivity in autism between thalamus and sensory regions such as the right postcentral gyrus and paracentral lobule involving the precentral gyrus, while evaluating the transient aspects of resting state functional connectivity. The current findings of hyperconnectivity between the right thalamus and pre and postcentral gyrus, sensory motor regions, are in line with previous investigations by Ayub et al. (2021), which demonstrated similar findings in autism compared with NT. The present study along with others investigating thalamus connectivity to sensorimotor cortical regions (Nair et al., 2013, 2015; Cerliani et al., 2015; Woodward et al., 2017; Ayub et al., 2021) suggest that a hyper connected profile of autistic brain may underlie the differences in sensorimotor processing seen in autistic individuals. These findings align with another study of a large sample of autistic participants that reported hyperconnectivity of the thalamus with the sensorimotor cortex, prefrontal cortices, and the temporal lobe. This was attributed to general cortical excitability differences and sensorimotor symptoms in autistic, compared to non-autistic, individuals (Woodward et al., 2017).

Thus, it should be noted that sensorimotor differences seem to be central to the characteristics of autism, such as differences in praxis and grip force (MacNeil and Mostofsky, 2012; Wang et al., 2015). A core feature of autism is restricted/repetitive behaviors, such as motor repetitions like hand flapping, self-stimulating behaviors, and manipulating objects, which can be calming to autistic individuals (Caldwell-Harris, 2021). These repetitive motor behaviors remain present in many autistic adults (Kapp et al., 2019). Atypical somatosensory input, such as tactile defensiveness is also seen in autism (Cascio et al., 2015). Most studies on sensory processing focus on children; however, a study on an autistic adult sample found that unusual sensory processing extends well into adulthood (Crane et al., 2009). Yet, other symptoms, such as poor visual/spatial skills have been reported in autistic children compared to their peers (Zhang et al., 2020). The current findings demonstrated widespread hyperconnectivity of the thalamus to cortical regions. Consistent findings from previous studies, together with the findings of our study, emphasize the strategic role of the thalamus in modulating sensorimotor signaling in the cortex. Previous studies have portrayed the thalamus as a critical functional hub for relaying sensory information to the cortex and for controlling motor signals (Hwang et al., 2017). Thalamocortical connectivity has been found to be associated with autism symptoms in both autistic and non-autistic adults (Ayub et al., 2021). The converging results support and extend previous observations regarding hyperconnectivity between thalamic and sensory regions.

We found that thalamic hyperconnectivity was related to an increase of severity in autistic traits as measured by the AQ. Analyses of brain-behavior associations using AQ have provided a significant relationship of thalamocortical connectivity and autistic traits. It appears that hyperconnectivity of the thalamocortical pathway predicts autistic symptoms. Autism and ADHD are neurodevelopmental disorders that often (50%–70% in autistic individuals) co-occur (Hours et al., 2022) and exhibit an overlap in symptom profiles regarding attention, performance monitoring, face processing, and sensory processing (Lau-Zhu et al., 2019). It may be hard to differentiate between these disorders in a clinical setting, especially measures like AQ, which may be limited in differentiating certain symptom profiles (Sizoo et al., 2009). Hence, connectivity patterns in thalamocortical pathways may provide insights into the mechanisms underlying autistic traits. Although the ADHD participants in the current study have lower AQ scores, the ADHD participants who had higher AQ scores exhibited greater connectivity between the left thalamus and the right precentral gyrus, and the right thalamus and the right precentral gyrus and the right pars opercularis (see Figure 3). Hyperconnectivity between these regions may contribute to autistic traits, such as difficulty in attention skills and differences om visuospatial perception (Baron-Cohen et al., 2001). Thus, it should be noted that among neurotypical and developmental disorders participants, thalamocortical hyperconnectivity seems to be specifically related to autistic symptoms.

### Limitations and future directions

The present study provides valuable new insights on thalamus connectivity during resting state in young adult autistic and ADHD participants. One limitation of the study is the relatively smaller sample size; however, the unique dataset was collected by our lab, which are autistic, ADHD, and NT participants who underwent the same protocol of testing and imaging (Bednarz et al., 2021, 2022; Svancara et al., 2022; Karavallil Achuthan et al., 2023). Although the sample size is smaller, it should be noted that none of the participants in the autistic group have a comorbid diagnosis of ADHD, nor did the ADHD group have a comorbid diagnosis of autism. Additionally, our study protocol had strict medication guidelines that all eligible participants must have been able to adhere to.

Regarding new directions, including younger participants may provide an opportunity to examine the developmental profile of thalamic connectivity. Future studies may explore thalamus connectivity in younger autistic children and in those with co-occurring autism and ADHD. Longitudinal studies with larger sample sizes in each group may elucidate more insights on the transient changes in connectivity as a function of brain development. Future studies could analyze hyperconnectivity of the thalamus to motor, somatosensory, and visuospatial cortices along with behavioral assessments and evaluations that measure the severity of symptoms such as motor planning, aversive reaction to tactile stimuli, and visual spatial skills. This could potentially help in planning interventions and treatment strategies in neurodevelopmental disorders.

## Conclusion

The present study demonstrates that the thalamic response in autism is significantly correlated with a wide range of cortical areas. There is a significantly stronger thalamic connectivity in the autistic participants when compared to ADHD and neurotypical participants, such as increased connectivity with right precentral/motor areas in both the left and right thalamus. Overall, this study found hyperconnectivity of thalamus in autism with various sensory areas of the brain. The present findings affirm the strategic role of thalamus in relaying sensory information to motor, somatosensory, and visuospatial areas which may provide insights into differences in sensory sensitivity in autistic individuals. Considering the limited number of studies focusing on thalamic connectivity in autism and ADHD, the findings of the current study provide valuable information on thalamic functional connectivity in neurodevelopmental disorders.

## Data availability statement

The original contributions presented in the study are included in the article/Supplementary material, further inquiries can be directed to the corresponding author.

## Ethics statement

The studies involving humans were approved by University of Alabama at Birmingham Institutional Review Board. The studies were conducted in accordance with the local legislation and institutional requirements. The participants provided their written informed consent to participate in this study.

## Author contributions

SK: Data curation, Formal analysis, Writing – original draft. DS: Conceptualization, Methodology, Resources, Writing – review & editing. PA: Writing – original draft, Writing – review & editing. CV: Writing – original draft, Writing – review & editing. HH: Conceptualization, Investigation, Methodology, Writing – review & editing. RK: Conceptualization, Funding acquisition, Project administration, Resources, Supervision, Writing – review & editing.
