# Supplementary material for: Thalamic functional connectivity and sensorimotor processing in neurodevelopmental disorders
Source: Front Neurosci. 2023 Dec 15;17:1279909. doi: 10.3389/fnins.2023.1279909 (PMC10755010; doi:10.3389/fnins.2023.1279909)

**Supplementary Materials**

**Supplementary Table S1.** Between subject 2x2 ANOVA to analyse the main effects of the three groups with left and right thalamus as the seed regions and by controlling the mean head motion with a voxel threshold, p =0.001 (uncorrected); cluster threshold, p =0.005 (familywise error corrected). Peak regions obtained after the seed-to-voxel analysis, MNI coordinates and cluster size are presented.

| Seed | MNI Coordinate | Peak regions | Cluster size |
| --- | --- | --- | --- |
| ***Right thalamus*** | +36 -38 +54 | **SPL-r, Postcentral Gyrus right** | 299 |
|  | +54 +6 +38 | **Precentral Gyrus right, Inferior Frontal Gyrus, pars opercularis Right** | 275 |
|  | +56 -10 +50 | **Postcentral Gyrus right, Supramarginal Gyrus, anterior division Right** | 197 |
|  | -52 -28 +42 | **Postcentral Gyrus Left, Precentral Gyrus Left** | 141 |
|  | -52 -10 +30 | **Postcentral Gyrus Left, Supramarginal Gyrus, anterior division Left** | 127 |
|  | -52 -4 +42 | **Precentral Gyrus Left** | 180 |
| ***Left thalamus*** | +56 +00 +44 | **Precentral gyrus right** | 66 |

**Supplementary Figure S1.** Unthresholded maps of right and left thalamus. A) Right thalamus; B) Left thalamus.


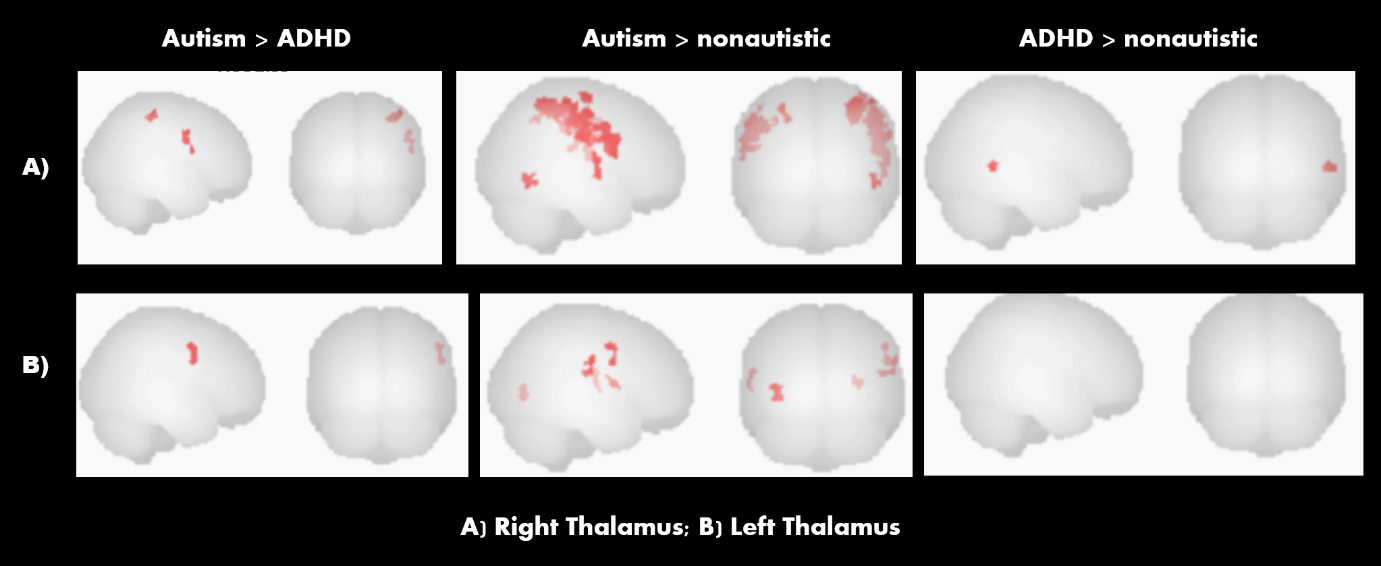


**Supplementary Figure S2.** A) Contrast Autism > ADHD, left thalamus as seed: linear regression plot of the connectivity values from the right precentral gyrus with the mean motion from autistic participants; B) the contrast Autism > ADHD, right thalamus as seed; the linear regression plot of the connectivity values from right post central gyrus and right superior parietal lobule with the mean motion from autistic participants; C) the contrast Autism > ADHD, left thalamus as seed: linear regression plot of connectivity values from right precentral gyrus with the mean motion from ADHD participants; D) shows the contrast Autism > ADHD, right thalamus as seed: linear regression plot of connectivity values from right post central gyrus and right superior parietal lobule with the mean motion scores from ADHD participants.  The Pearson correlation coefficient obtained for each contrast for the selected seeds are given inside the regression plot.


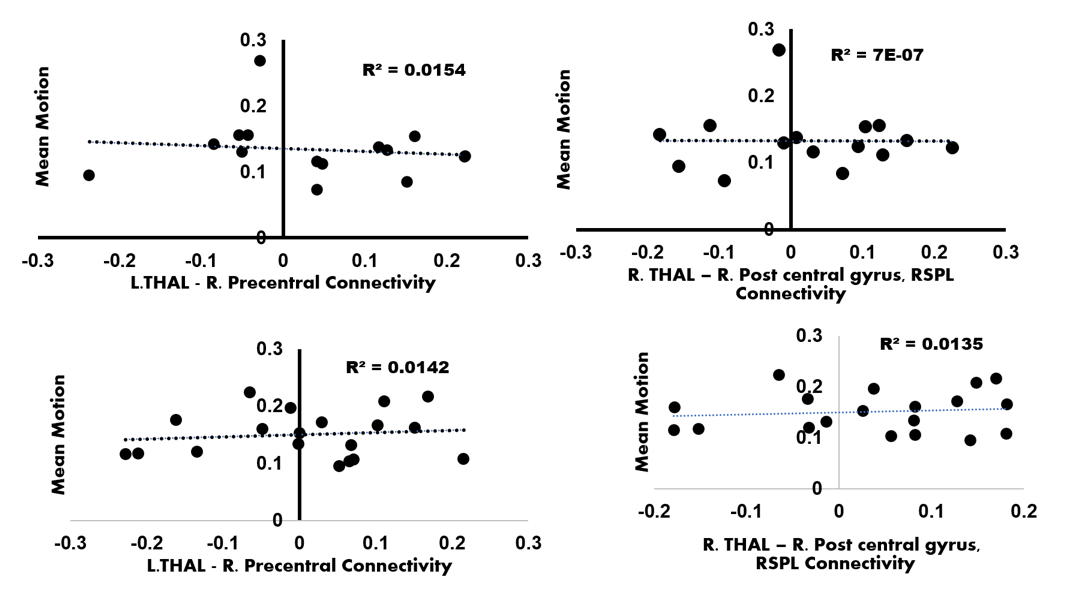

Supplement: Supplementary file 1 [file Data_Sheet_1.docx]
